# Case of Spinal Apoplexy

**Published:** 1831-04-01

**Authors:** 


### MILITARY HOSPITAL OF NAMUR.

### CASE OF SPINAL APOPLEXY. By M. FALLOT, M.D.

THE following case is interesting in many points of view, as will be seen in its perusal.

A man, 20 years of age, tall and well-formed, having never before experienced illness, began to complain of loss of appetite and muscular force, to which was added occasional hæmoptysis of two or three days duration at a time. Nevertheless he continued his military duties till the 11th of May, when he entered the hospital, in the following condition:—face flushed, swelled, and puffy under the eyes, which were injected—complained of slight headache, with extreme lassitude, pains in his limbs, and some difficulty of swallowing. The head was clear, the pulse not accelerated, skin natural, tongue moist, breathing easy. In the evening, however, there were decided symptoms of cerebral congestion, and he was bled to 14 ounces, after which the cerebral symptoms were much mitigated. The night was passed in a state of sleeplessness and restlessness. On the visit next morning, the intellects were clear; but there was great inquietude and jactitation, in consequence, as he said, of the intolerable pains in his limbs, which were spasmodically affected from time to time, during which, his face became of a blue colour, his head was strongly retracted backwards, and any attempt to bring it forward, was attended with violent pain. The cervical region was swelled on the posterior part, and very hot. The pulse was concentrated—the bowels confined. Forty leeches were applied to the nape of the neck, and a purgative lavement exhibited. By these means, the cervical pain and tumefaction were diminished—and also the jactitation and pain in the limbs. At the evening visit, the patient was calm—he could bring his head more forward—deglutition was easy—the bowels were well opened—there were colicky pains—and ten leeches were applied in the night to the track of the colon. 13th May. There were symptoms of pulmonary engorgement, as râle crepitant—cough—sero-sanguinolent expectoration—blueness of the face—breathing short and laborious—action of the heart violent and tumultuous—pulse thready—extremities cold—cessation of the spasmodic contractions. We need not pursue the symptomatology. He died the same afternoon, at 4 o'clock.

*Post-mortem Examination.* The vessels of the brain were exceedingly distended, and the arachnoid infiltrated. The cerebral substance itself was firm, and unaltered. The vessels of the spinal membranes were also extremely injected. A layer of blood was found opposite to the 5th cervical nerves beneath the pia mater of the medulla, which was thereby raised. This layer gradually increased in thickness and breadth, as it descended, and covered the whole medulla spinalis down to the cauda equina. No morbid appearance presented itself in the substance of the spinal marrow itself. The lungs were gorged with black and frothy blood. The heart was very large, and the parietes of the ventricles of unusual thickness.

This kind of case is not common, and deserves record. The symptoms were signally alleviated by the detractions of blood; and had the effusion on the spinal marrow not been so rapid, it is probable that life might have been saved.—*Journal Complementaire.*

